# Co-Microencapsulation of Cushuro (*Nostoc sphaericum*) Polysaccharide with Sacha Inchi Oil (*Plukenetia huayllabambana*) and Natural Antioxidant Extracts

**DOI:** 10.3390/antiox13060680

**Published:** 2024-05-31

**Authors:** Nancy Chasquibol, Axel Sotelo, Mateo Tapia, Rafael Alarcón, Francisco Goycoolea, María del Carmen Perez-Camino

**Affiliations:** 1 Grupo de Investigación en Alimentos Funcionales, Carrera de Ingeniería Industrial, Instituto de Investigación Científica, Universidad de Lima, Av. Javier Prado Este 4600, Fundo Monterrico Chico, Surco, Lima 15023, Peru; aasotelo@ulima.edu.pe (A.S.); mtapiac@ulima.edu.pe (M.T.); ralarcor@ulima.edu.pe (R.A.); 2 School of Food Science and Nutrition, University of Leeds, Leeds LS2 9JT, UK; f.m.goycoolea@leeds.ac.uk; 3 Faculty of Biology, University of Murcia, Campus de Espinardo, 30100 Murcia, Spain; 4 Instituto de la Grasa-Consejo Superior de Investigaciones Científicas, Campus Universidad Pablo de Olavide Ed. 46, Crtra. Sevilla-Utrera km 1, 41013 Sevilla, Spain; mcperezcamino@ig.csic.es

**Keywords:** antioxidant extracts, co-microencapsulation, cushuro, *Nostoc sphaericum*, polysaccharide

## Abstract

Cushuro (*Nostoc sphaericum*) polysaccharide was used to co-microencapsulate sacha inchi oil, natural antioxidant extracts from the oleoresin of charapita chili peppers (*Capsicum frutescens* L.) and grape orujo (*Vitis vinifera* L.). Encapsulation efficiency, moisture, particle size, morphology, oxidative stability, shelf-life, solubility, essential fatty acid profile, sterol content and antioxidant capacity were evaluated. The formulations with grape orujo extract showed higher oxidative stability (4908 ± 184 h), antioxidant capacity (4835.33 ± 40.02 µg Trolox/g ms), higher phenolic contents (960.11 ± 53.59 µg AGE/g ms) and a smaller particle size (7.55 µm) than the other formulations, as well as good solubility and a low moisture content. Therefore, grape orujo extracts can be used as natural antioxidants. The fatty acid composition (ω-3) remained quite stable in all the formulations carried out, which also occurred for sterols and tocopherols. In combination with gum arabic, grape orujo extract offered oxidative protection to sacha inchi oil during the first week of storage.

## 1. Introduction

Algae and microalgae present bioactive compounds such as polysaccharides, proteins, lipids, and polyphenols, with polysaccharides being the most abundant [1]. Polysaccharides are macromolecules of sugars which are linked by glycosidic bonds, which present positive health benefits because they show antitumor, anticoagulant and antiviral activities [2]. Polysaccharides also exhibit antioxidant activity to counteract diseases caused by oxidative stress such as diabetes, obesity, hepatitis and cancer [3]. These compounds are used in the food industry as additives to homogenize, increase the shelf-life of foods and reduce production costs by modifying the rheology of complex food systems [4,5]. In addition, they are used as encapsulating agents to protect bioactive compounds of functional interest. Currently, commercial biopolymers used as food additives include gum arabic, guar gum, maltodextrin and pectin, among others [6].

Applications of algal polysaccharides in microencapsulation processes have been previously reported. Agar fractions extracted from the red alga Gelidium were applied as a microencapsulating agent for *Bifidobacterium pseudocatenulatum* [7]. Kavoosi et al. [8] showed that agar, alginates, and carrageenan are important wall materials for the microencapsulation of Zataria essential oil. Microencapsulated beta cells (INS1E) with fractions of fucoidan, a sulfated polysaccharide, were obtained from brown algae [9]. Wu et al. [10] evaluated the rheological properties of tara gum, concluding that it exhibits higher viscosity than elastic capacity.

The cushuro (*Nostoc sphaericum*) is a viscous spherical microalga that forms both microscopic and macroscopic colonies in various marine environments and in high Andean areas of Peru (>3000 masl). In Peru, it is found in the departments of Ancash, Junín, Cajamarca, Huánuco, Cusco and Puno, where there are lagoons with crystalline waters rich in nitrogen, which favors its development; it is also known as murmunta, llullucha or llayta [11,12]. 

*Nostoc sphaericum* could be an important source of dietary fiber or a viscous additive in the manufacturing of food products [13]. Likewise, the polysaccharides in cushuro (*Nostoc sphaericum Vaucher ex Bornet and Flahaut*) presented hypoglycemic activity at concentrations of 50 mg/mL [14]. The incorporation of ultrasound-assisted extraction technology (of 540 W, temperature 353.15 K, time 25 min and solid:liquid ratio 1:50, g/mL) has been studied to obtain polysaccharide fractions from cushuro (*N. Commune*) [15]. Cushuro polysaccharides (*N. commune and N. Sphaericum*) was extracted to develop fruit nectars and evaluate their rheological properties [16]. 

The aji charapita pepper (*Capsicum frutences*) is a plant that grows in the Amazon region of Peru, Native to Iquitos. It is considered one of the hottest peppers in the country. It stands out for its high vitamin contents (A, C, E and K, along with B complex) [17] and presents phenolic compounds such as flavonoids and phenolic acid [18]. Capsaicinoids are alkaloids present in aji charapita peppers which provide the characteristic spicy flavor, [19] and due to their pungency, they are consumed in sauces, in macerates with lemon, in dehydrated form [20] and as an important ingredient in Peruvian gastronomy.

The wine industry demands a large production of grapes, which increases every year and generates skin and seed waste (bagasse or grape orujo) (*Vitis vinifera* L.). Wine production generates 30,000 MT/year of grape orujo in Peru [21], with Moquegua (15%) being the department with the highest production, resulting in a significant environmental impact. These residues stand out for their high contents of phenolic compounds (0.09–0.35%/g dry orujo), such as anthocyanins, flavonols and protoanthocyanidins [22], making them a potentially important source of phenolic compounds.

Chasquibol et al. [23] concluded that the co-microencapsulation process using antioxidant extracts from camu-camu (*Myrciaria dubia (HBK) Mc Vaugh*), Andean potato (*Solanum tuberosum andigenum*) and elderberry (*Sambucus peruviana*) fruit skins increased the shelf-life of sacha inchi (*Plukenetia huayllabambana*) oil from 1176 to 3116 h.

The objectives of the present research were to evaluate the co-microcapsulation process of cushuro (*Nostoc sphaericum*) polysaccharide with sacha inchi (*Plukenetia huayllabambana*) oil and antioxidant extracts from grape orujo (*Vitis vinifera* L.) and aji charapita peppers (*Capsicum frutences*) in terms of humidity, peroxide index, total and surface phenolic contents, DPPH antioxidant activity, fatty acid profile, oil encapsulation efficiency, solubility, particle size distribution, morphology and oxidative stability.

## 2. Materials and Methods

### 2.1. Raw Material

Cushuro (*Nostoc sphaericum*) was obtained from the lakes of Ututo (4420 masl), Tacash (4412 masl) and Llacsha (4512 masl) in the district of Cotaparaco, province of Recuay, department of Ancash in Peru. The cushuro was washed, dried at 60 °C for 12 h in an infrared dehydrator (IRD D18, Sevilla, Spain), ground in a food grinder (Grindomix GM200/Restch, Haan, Germany) to obtain cushuro flour and stored in aluminized bags at room temperature. All reagents used were of analytical grade and supplied by Merck, and Mili Q water was used.

Sacha inchi (*Plukenetia huayllabambana*) seeds were collected in the province of Rodriíguez de Mendoza, Amazon region, Peru. Sacha inchi oil was cold-pressed at the Functional Food Laboratory of the University of Lima, Peru, and stored at 4 °C in a dark flask. Grape orujo (*Capsicum frutences*) was collected from wine fermentation waste in the province of Cañete, department of Ica-Peru, and aji charapita peppers (*Vitis vinifera* L.) were obtained from a local market in the city of Lima, Peru. Grape orujo and aji charapita peppers were washed and dried in the infrared dehydrator (IRC DI8, Sevilla, Spain) at 40 °C, ground in the food grinder (Grindomix GM200/Restch) and stored in polyethylene bags at room temperature until further use. Gum arabic (GA) and maltodextrin (MD) were purchased from Frutarom, Peru S.A. 

Cushuro (*Nostoc sphaericum*) polysaccharide was obtained at the Functional Food Laboratory of the University of Lima. The cushuro flour was dispersed in water, then stirred for 30 min at 80 °C and filtered under vacuum. The solid residue was re-liquefied with water and the extraction process was repeated a second time. The supernatants were filtered under vacuum with a muslin cloth, and the final filtrate was concentrated using a rotary evaporator (Buchi B-100, Flawil, Switzerland) and precipitated with isopropanol to a final concentration of 70% alcohol. The precipitate obtained was washed with 80, 90 and 99% isopropanol and dried at 50 °C for 3 h. It was then ground in a mortar and stored in aluminized bags until further use.

### 2.2. Co-Microencapsulation Process of Cushuro Polysaccharide

#### 2.2.1. Formulations

Table 1 shows six formulations developed with cushuro (*Nostoc sphaericum*) polysaccharide (CP), sacha inchi (*Plukenetia huayllabambana*) oil (SIOPH), gum arabic (GA) and maltodextrin (MD) as encapsulating agents according to the procedures described by Chasquibol et al. [23], with some modifications made after our preliminary tests. Cushuro polysaccharide was dispersed in ultrapure water at 80 °C at a concentration of 0.6% (*w*/*v*) for 3 h. After cooling the solution, the encapsulating materials were hydrated in the polysaccharide solution under constant stirring on a pedestal stirrer (DLAB OS20-S, DLAB Scientific Co., Beijing, China) overnight, then sacha inchi oil (*Plukenetia huayllabambana*) was added to form the oil-in-water (o/w) emulsion in the Silverson homogenizer (L5M-A, Silverson, Chesham, Buckinghamshire, UK) at 9000 rpm for 10 min in a cold-water bath to protect the sacha inchi oil from lipid oxidation.

#### 2.2.2. Co-Microencapsulation of the Cushuro Polysaccharide with Sacha Inchi Oil and Antioxidant Extracts

Eight formulations (Table 2) were prepared using cushuro polysaccharide (*Nostoc sphaericum*) (CP), with sacha inchi oil (*Plukenetia huayllabambana*) (SIOPH), natural antioxidant extracts from grape orujo (*Vitis vinifera* L.) (GOE), aji charapita (*Capsicum frutences*) pepper (ACHPE) and commercial antioxidant (BHT). Gum arabic (GA) and maltodextrin (MD) were used as encapsulating agents according to a method previously reported [23].

Cushuro polysaccharide (CP) was dispersed in ultrapure water at 80 °C at a concentration of 0.6% (W/V) for 3 h. After cooling the solution, gum arabic (GA) and maltodextrin (MD) were dispersed in the polysaccharide solution under constant stirring overnight. The antioxidant extracts of grape orujo (GOE) (200 ppm) and Ají Charapita pepper (ACHPE) (200 ppm) were added according to the oil content in each formulation. The concentration of total solids was adjusted to 30%. Sacha inchi oil (*Plukenetia huayllabambana*) was added at a 33.33% concentration (1 g oil/3 g encapsulating agent) of total solids. A Silverson homogenizer (L5M-A, Silverson, Chesham, Buckinghamshire, UK) was used to obtain homogeneous emulsions at 9000 rpm for 10 min using a cold-water bath to prevent the temperature of the emulsion from exceeding 25 °C. The spray drying conditions were detailed by Chasquibol et al. [24], with some modifications. Spray dryer equipment (Büchi B-290, Büchi Labortechnik AG, Flawil, Switzerland) was used, with inlet and outlet temperatures of 140 and 70 °C, respectively, and a feed flow rate of 55 mL/min. The samples obtained were stored in aluminized bags at room temperature until further analysis.

#### 2.2.3. Moisture

The moisture of the microcapsules was determined using a halogen balance model KERN DBS 60-3. A quantity of 1 g of microcapsules was placed on the pan of the halogen balance and the analyzer started heating by means of the built-in halogen lamp. The balance registered weight loss values until reaching a constant value corresponding to the moisture content of the weighed sample [25].

#### 2.2.4. Peroxide Index (PI)

The peroxide value was determined by iodometric titration [26]. Two grams of microcapsules was dissolved in 25 mL of chloroform: glacial acetic acid (3:2 *v*/*v*) and vortexed for 30 s. Of a saturated potassium iodide solution, 1 mL was then added. After 5 min in the dark, 75 mL of distilled water was immediately added, and the titration was started. The released I_2_ was titrated with Na_2_S_2_O_3_ (0.01 N), using a starch solution (1%) as an indicator, until the solution became colorless (American Oil Chemists Society, 2009) [27].

#### 2.2.5. Total Phenolic Content (TPC) and Surface Phenolic Content (SPC)

The total phenolic content (TPC) of the microcapsules was determined by the Folin–Ciocalteau method [28] with some modifications. The microcapsules (15 mg) were dissolved in 4.5 mL of methanol and then shaken for 1 min in a vortex (VELP Scientifica, Usmate Velate, Italy). Of Folin–Ciocalteau’s reagent (0.2 N), 2.5 mL were added with stirring for 1 min using a vortex. After 5 min, 2 mL of a sodium carbonate solution (20%) were added, mixed and kept in a water bath for 20 min at 80 °C. The mixture was cooled and then filtered through Whatman No. 2 filter paper. The absorbance of the solution was measured at 760 nm using a spectrophotometer (Shimatzu UV-1280, Kyoto, Japan). Ultrapure water was used as a control blank. The results are expressed as µg gallic acid equivalent (GAE) per gram of microcapsules. All analyses were performed in triplicate and the results are expressed as mean values. For the determination of surface phenolic content (SPC), 24 mg of microcapsules was dissolved in 4.5 mL of methanol and vortexed for 1 min, then filtered through Whatman N°2 filter paper. The surface phenolic content was measured according to the same method described for TPC. The encapsulation efficiency percentage of polyphenol microencapsulation was calculated using Equation (1):

Encapsulation efficiency percentage (EEP) (%) = ((TPC − SPC))/TPC × 100(1)

#### 2.2.6. Determination of Antioxidant Activity via Radical DPPH

Antioxidant activity was determined by the DPPH method [28] with some modifications. Of the microcapsules, 15 mg was dissolved in 4.5 mL of methanol/acetic acid/water (50:8:42, *v*/*v*/*v*/*v*/*v*), then shaken for 1 min and rested in a water bath for 20 min at 80 °C. This content was mixed with 3.9 mL of a DPPH radical solution at 25 ppm (2.5 mg of DPPH in 100 mL of methanol) and kept in the dark at 25 °C. The mixture was vortexed for 1 min and then filtered through Whatman No. 2 filter paper. The absorbance of the samples was measured at 517 nm after 1 h of incubation in the dark. For the control sample (blank), 500 µL of methanol were mixed with 3.9 mL of the DPPH radical solution at 25 ppm and left in the dark at 25 °C. The absorbance (Abs517 control) was measured at 517 nm using a spectrophotometer (Shimatzu UV-1280, Kyoto, Japan). All analyses were performed in triplicate. The percentage inhibition (% I) of free radicals was calculated according to Equation (2):

(%I) = [(Abs517 Control) − (Abs517 Sample)/(Abs517 Control)] × 100(2)

#### 2.2.7. Free Oil (Not Encapsulated) and Total Oil

The fraction of free or unencapsulated oil was quantitatively determined by stirring with hexane at room temperature for a controlled time, according to the method reported by Sankarikutty et al. [29]. Of hexane, 10 mL was added to 1 g of microcapsules and gently agitated for 10 min at room temperature. The extract was immediately filtered over 0.45 mm and 0.25 mm diameter PTFE filter, and the solvent was evaporated by vacuum at a rotary evaporator and dried to constant weight using a stream of nitrogen.

The total oil extraction procedure was based on a method previously reported [30]. Forty mL of deionized water at 65 °C was added to 5 g of microcapsules. After slight agitation, 8 mL of NH_4_OH at 30% (*w*/*w*) was added and agitated for 15 min while maintaining the temperature at 65 °C. The mixture was then allowed to cool to room temperature and the extraction was carried out. Three liquid–liquid extractions were used with the following solvent mixtures: first extraction: 20 mL ethanol, 50 mL diethyl ether and 50 mL hexane; second extraction: 10 mL ethanol, 25 mL diethyl ether and 25 mL hexane; third extraction: 25 mL diethyl ether and 25 mL hexane. Vigorous shaking was performed between one solvent and the other and in the case of persistent emulsion, a few milliliters of ethanol were added. The three mixed extracts were filtered through filter paper containing anhydrous Na_2_SO_4_. The solvent was then removed under vacuum using a rotary evaporator at room temperature. On completion, it was brought to constant weight using a stream of nitrogen. 

Once the microencapsulated oils were obtained, their fatty acid, tocopherol and sterol compositions were determined.

#### 2.2.8. Fatty Acid Profile

Fatty acids methyl esters (FAMEs) were made according to procedures previously reported [31,32]. Of each extracted oil (as specified in Section 2.2.7), 50 mg was weighed into a 4 mL capacity test tube with a screw cap. Next, 2 mL of heptane and 0.5 mL of 2N KOH in methanol were added. After shaking and phase separation, the upper phase was transferred to a chromatography vial and injected into the gas chromatograph (GC).

The FAMEs formed were analyzed using an Agilent 7890B gas chromatograph (Agilent Technologies, Santa Clara, CA, USA) equipped with an SP2380 polar capillary column (poly (90% biscyanopropyl-10% cyanopropylphenyl) siloxane, 60 m × 0.25 mm id; 0.20 µm film thickness) and a flame ionization detector (FID). The injector and detector temperatures were 225 and 250 °C, respectively. Hydrogen was used as a carrier gas at a flow rate of 1.0 mL/min. The oven temperature was set at 165 °C and increased to 230 °C at a rate of 3 °C per min. This temperature was maintained for 2 min. The injection volume was 1 μL.

#### 2.2.9. Tocopherol Analysis by HPLC

High-performance liquid chromatography (HPLC), using a silica column and fluorescence detector, was used to quantitatively determine the tocopherols present in the oil samples extracted from the microencapsulated oils, as well as from the sacha inchi oil (*Plukenetia huayllabambana*). A mixture of hexane:isopropyl alcohol (99:1) was used as an eluent [33]. Thirty mg of oil was weighed, made up to the mark with chromatographic-quality hexane in a 10 mL volumetric flask and analyzed immediately. The wavelengths were 290 nm and 330 nm for excitation and emission, respectively.

#### 2.2.10. Analysis of Sterols by GC

The simplified method published by Garcia-Gonzalez et al. [34] was followed with slight modifications. Of oil, 1 g ± 1 mg of was weighed and 50 μL of the internal standard α-cholestanol (1 mg/mL) was added. After evaporating the solvent, 5 mL of 3% (*w*/*v*) sodium methylate was added and kept at 80 °C for 30 min. Next, a few drops of phenolphthalein and a mixture of sulfuric in 4% (*v*/*v*) methanol were added in sufficient quantity until transparency of the samples, which were then kept for another 30 min at 80 °C. After cooling the sample, 3 extractions were made with 2 mL portions of hexane. These extractions were washed twice with 2 mL of distilled water and the hexane fraction was passed through a filter containing anhydrous sodium sulfate. The solvent was removed by rotary evaporation and then the lipid sample was derivatized with 250 μL of the mixture: BSTFA-TMCS:Pyridine 1:1 (*v*/*v*). One μL of the sililated sample was injected into a gas chromatograph equipped with a 30 m × 0.25 I.D. × 0.1 μm film thickness HP-5 column. The oven temperature was maintained at 260 °C throughout the analysis, and injector and detector temperatures at 300 °C.

#### 2.2.11. Solubility

Solubility was determined by dissolving 0.5 g of microcapsule in 20 mL of distilled water in a 50 mL volumetric flask. It was then vortexed for 5 min and centrifuged at 3000 rpm for 5 min. Of the supernatant, 20 mL was taken and placed in a vessel which was heated at 105 °C for 2 h. Solubility (%) was calculated according to the weight difference [24].

#### 2.2.12. Particle Size Distribution and Microcapsule Morphology

The particle size distribution and morphology of the microcapsules were determined using a scanning electron microscope (Zeiss, EVO-MA10, Dublin, CA, USA). Using an aluminum sample holder, a carbon ribbon was placed on top of the microcapsule to be analyzed. The prepared samples were placed in the scanning electron microscope chamber for recording De Broukere D [4,3] mean diameter or volume-weighted mean size and the micrographs.

#### 2.2.13. Oxidative Stability

The oxidative stability of the microcapsules was evaluated using the Rancimat method [24] on an 892 Professional Rancimat© (Metrohm, Herisau, Switzerland). The induction period (IP), defined as the time required to produce a marked increase in conductivity at the temperatures of 70, 80, 90 and 100 °C was determined with an air flow rate of 20 L/h. All determinations were performed in triplicate. The extrapolation of the lifetime for all samples at 25 °C was calculated using the equipment software according to Equation (3):

Shelf-life = A × e^(B × T)(3)

where T is the temperature at which the induction period is calculated. A and B are the regression coefficients based on the determinations of IP.

### 2.3. Statistical Analysis

The results for oil and antioxidant encapsulation efficiency, moisture, oxidative stability, shelf-life, solubility and essential fatty acid profile are presented as the mean ± standard deviation. All assays were carried out in duplicate or triplicate. An analysis of variance (ANOVA) and Tukey’s test were performed at 95% significance level with Minitab 19 (Minitab^®^ statistical 19 software, State College, PA, USA).

## 3. Results and Discussion

### 3.1. Sample Viscosity

Table 3 reports the viscosity values for the samples. As can be observed, with an increasing polysaccharide concentration (0.35–0.45%) the dynamic viscosity increased, due to the ability of cushuro polysaccharide to form high-viscosity solutions at low concentrations [35]. The maximum viscosity value allowed for drying by the spray dryer (Büchi B-290-Switzerland) was 300 cp and according to the results, samples 2, 3, 5 and 6 present viscosity values above the maximum allowed for viscosity. For this reason, samples 1 and 4 with the lowest percentage of polysaccharide were selected.

Jurado et al. [13] studied the viscosity of the polysaccharides of *Nostoc commune* and *Nostoc sphaericum* species at 2% (*w*/*v*) in water, obtaining values of 4.5 cp and 38.3 cp, respectively. Yupanqui et al. [36] determined the viscosity of the polysaccharides of *Nostoc commune* and *Nostoc sphaericum* species, with the hydrocolloid of *N. sphaericum* species dried by lyophilization being the one that presented higher viscosity at 7 °C and at 0.25% with a value of 118.3 cp, surpassingly four times greater than the hydrocolloid of *N. commune* species dried by hot air with a low viscosity of 28.0 cp under the same evaluation conditions. The emulsion viscosity also affected the spray drying rate through the formation of large and elongated droplets, which negatively affected the drying rate [37].

### 3.2. Moisture Content and Peroxide Index (PI)

According to Table 4, the moisture contents of the formulations (4.04–4.71%) were similar to those previously reported [23] (2.59–7.59%) and are in the range established for dehydrated foods (<10%). The low moisture content is due to the temperature of the drying process [38]. However, in spray-dried products, it is recommended that the moisture content should be <4% [39,40]. The higher moisture content was probably due to the high hygroscopicity of gum arabic and cushuro polysaccharide, which present hydrophilic ramifications in their structure and favor a higher absorption of water from the environment [41,42].

Table 4 shows the results of the peroxide value of the microcapsule formulations. The PI content for formulations (3–6) with natural antioxidants ranged from 3.04 ± 0.10 to 3.62 ± 0.30 meq O_2_/kg oil. These values were lower than the PI of formulations (7–8) with commercial antioxidant BHT (3.50 ± 0.28–3.66 ± 0.11 meq O_2_/Kg oil).

The antioxidant extracts of grape orujo and aji charapita pepper could be used to replace the commercial antioxidant BHT and offer protection against the oxidation of sacha inchi oil (*P. huayllabambana*). The co-microencapsulation of fish oil (tuna) using chitosan and maltodextrin without antioxidants presented a PI (2.94 ± 0.04 meq/Kg oil) which was lower than the PI obtained for formulations 1 and 2 [43]. 

### 3.3. Total Phenolics Content (TPC), Surface Phenolic Content (SPC) and Encapsulation Efficiency (% EEP)

The TPC values are shown in Table 5. Formulation 3 with natural grape orujo antioxidant (CP + SIOPH + GOE (200 ppm) + GA + MD) (960.11 ± 53. 59 µg GAE/g ms) presented higher TPC than the formulations with the commercial antioxidants BHT: 7 (CP + SIOPH + BHT (200 ppm) + GA) (790.71 ± 38.87 µg GAE/g ms) and 8 (CP + SIOPH + BHT (200 ppm) + GA + MD) (628.46 ± 50.30 µg GAE/g ms). Formulation 3 presented a lower SPC concentration (18.48 ± 2.86 µg GAE/g ms) and higher percentage of EEP (%) due to the fact that grape orujo extract presents a higher content of phenolic compounds such as gallic, caftaric, chlorogenic acid, among others [44]. Nevertheless, formulation 2, without antioxidants, presented the lowest EPP value (%) (87.19 ± 1.59%), due to the higher SPC content (33.04 ± 5.34 µg GAE/g ms) than the other formulations.

Formulations 5 (CP + SIOPH + ACHPE (200 ppm) + GA) (661.79 ± 77.18 µg GAE/g ms) and 6 (CP + SIOPH + ACHPE (200 ppm) + GA + MD) (490.52 ± 64.05 µg GAE/g ms) with aji charapita pepper extract presented a lower TPC content than formulations with grape orujo and BHT. However, they presented higher TPC concentration than formulations without antioxidants 1 and 2. According to Meckelmann et al. [45], capsicum pepper species are an important source of vitamin C and phenolic compounds, especially quercetin, which would explain the high TPC in the formulation with aji charapita pepper. On the other hand, the cushuro polysaccharide also presented phenolic compounds and flavonoids [46] that would also have contributed to the TPC results in all formulations.

### 3.4. Antioxidant Activity of Microcapsules

As shown in Table 5, formulations 3 (CP + SIOPH + GOE (200 ppm) + GA) (4835.33 ± 40.02 µg Trolox/g ms) and 5 (CP + SIOPH + ACHPE (200 ppm) + GA) (4312.72 ± 23. 31 µg Trolox/g ms) with natural antioxidants, presented higher antioxidant activity than the formulations with the commercial antioxidant BHT 7 (CP + SIOPH + BHT (200 ppm) + GA) (4641.18 ± 11.18 µg Trolox/g ms) and 8 (CP + SIOPH + BHT (200 ppm) + GA + MD) (3428.41 ± 11.06 µg Trolox/g ms). These results are due to the high content of phenolic compounds in the aji charapita pepper and grape orujo [47,48].

The formulations without antioxidants, 1 (CP + SIOPH + GA) (3169.41 ± 35.23 µg Trolox/g ms) and 2 (CC + SIOPH + GA + MD) (2522.68 ± 61.27 µg Trolox/g ms), presented a lower antioxidant activity content than the natural extracts of grape orujo and aji charapita pepper.

### 3.5. Freel Oil (Not Encapsulated) and Total Oil

In relation to free oil, formulations 5 (CP + SIOPH + ACHPE (200 ppm) + GA) and 6 (CP + SIOPH + ACHPE (200 ppm) + GA + MD) with the antioxidant extract of aji charapita pepper presented higher values (10.79 ± 0.36–14.10 ± 0.49%) than the other formulations (6.52 ± 0.21–9.70 ± 0.66%) (Table 6), with or without maltodextrin, which indicated the lower encapsulation efficiency for the formulations containing aji charapita pepper extract.

The procedure for obtaining the total encapsulated oil was tedious and emulsions were easily formed during extraction. Therefore, the recovery was not usually complete in many cases. The average value of the total oils obtained for the eight formulations was 17.2%, ranging from 14.5 to 22.1% and corresponding to formulations 2 (CP + SIOPH + GA + MD) and 6 (CP + SIOPH + ACHPE (200 ppm) + GA + MD), respectively.

### 3.6. Fatty Acid Profile

The results of the fatty acid compositions are shown in Table 7, where the columns, corresponding to the fatty acids, are in the same order as they appear in the chromatograms: C16:0, C16:1, C17:0, C17:1, C18:0, C18:1,C18:2, C20:0, C18:3, C20:1. It can be observed that the major fatty acid was ω3-linolenic (C18:3) at 57%, followed by ω6-linoleic (C18:2) (27%) and oleic (C18:1) (8–9%). For fatty acids with isomers of positions ω9 and ω7, such as palmitoleic and oleic acids, the data shown in the table are the sum of both isomers. The statistical analysis of the results showed that for one of the major fatty acids, linoleic acid (ω6), the differences found among all the encapsulation experiments were not significant. In the case of linolenic fatty acid (ω3), the differences among all the formulations with the initial oil were not significant, except for formulations 2 and 3. For the other fatty acids, although the statistical study showed that there was a difference between some of the samples, they were not relevant. The total *trans*-fatty values in all the samples studied was lower than 0.01%. In short, these data show us that the encapsulation processes carried out do not alter the fatty acid composition.

### 3.7. Unsaponifiable Minor Compounds: Tocopherols and Sterols

**Figure 1:**
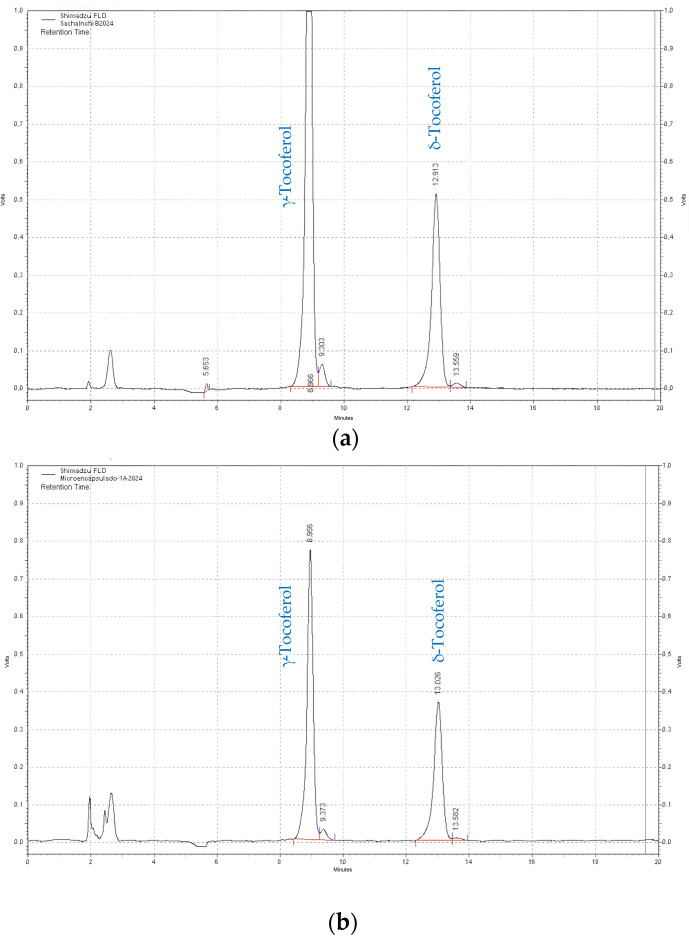
HPLC-FL chromatograms of tocopherols corresponding to the initial oil (**a**) and total oil (**b**) extracted from formulation 1 (CP + SIOPH + GA).

Gamma and delta are the two molecular species of tocopherols present in sacha inchi oils and in those extracted from microcapsules. The ratio between these two tocopherols is approximately 65:35, and in that order, they were present in the sacha inchi oil and in those extracted from the microcapsules. Figure 1a,b shows two chromatograms of tocopherols corresponding to the starting SIOPH and the total oil extracted from formulation 1 (CP + SIOPH + GA).

The quantitative data of total tocopherols of the eight formulations, as well as the initial SIOPH, are presented in Table 8, where the percentage of the two species present (γ-, and δ-tocopherol) and the ratio between them are specified. It should be noted that the majority value was obtained in the unencapsulated oil (1077.8 ± 7.1), and the losses due to encapsulation were between 19.2 and 10.0%, corresponding to formulations 1 and 3, respectively.

In relation to the data obtained for sterols, the profiles for all the oils obtained after the encapsulation process were similar to the initial SIOPH initial, with β-sitosterol, stigmasterol and campesterol as the major sterols. Table 9 shows their percentages as well as their total quantities using cholestanol as internal standard. The total sterols were higher than 2.000 ppm, except for sample 1 (1844.0 ppm). Compared to the SIOPH (not encapsulated), samples 2, 3, 4 and 5 showed no differences and samples 1, 6, 7 and 8 differed statistically from the rest, with values between 1844.0 and 2156.5 ppm. The stigmasterol/campesterol ratios were between 3.45 and 6.27, which are much higher than usual for edible seed oils.

### 3.8. Solubility

The solubility values are shown in Table 10. The formulations with natural antioxidants (3–6) presented higher solubility than the formulations without antioxidants (1–2), and similar solubility to the formulations with the commercial antioxidant BHT. The highest solubility percentages were observed in formulation 4 (CP + SIOPH + GOE (200 ppm) + GA + MD) (87.22 ± 0.63%) and formulation 6 (CP + SIOPH + ACHPE (200 ppm) + GA + MD) (86.02 ± 0.87%), followed by formulation 8 (CP + SIOPH + BHT (200 ppm) + GA + MD) (85.47 ± 0.47%). The addition of maltodextrin improved the solubility of the microcapsules due to its high solubility and low viscosity, thus allowing for a rapid release of bioactive compounds [49]. Solubility is the measure of the quality of food ingredients, so microcapsules that have good solubility properties are of superior quality [43]. 

### 3.9. Particle Size Distribution and Microcapsule Morphology

According to the particle size distribution (Table 10), D [4,3] values varied from 7.55 µm to 11.71 µm and were typical of particles obtained by spry drying [50]. The formulations without antioxidants 1 (CP + SIOPH + GA and 2 (CP + SIOPH + GA + MD) presented higher values for D [4,3] (µm) than the formulations with natural grape orujo antioxidants, 3 (CP + SIOPH + GOE (200 ppm) + GA) and 4 (CP + SIOPH + GOE (200 ppm) + GA + MD). Furthermore, the formulations without antioxidants presented similar D values [4,3], such as the formulations with natural grape orujo antioxidant, 5 (CP + SIOPH + ACHPE (200 ppm) + GA) and 6 (CP + SIOPH + ACHPE (200 ppm) + GA + MD) and the formulations with the commercial antioxidant BHT, 7 (CP + SIOPH + BHT (200 ppm) + GA) and 8 (CP + SIOPH + BHT (200 ppm) + GA + MD). In addition, a monodisperse distribution was observed in all formulations.

**Figure 2:**
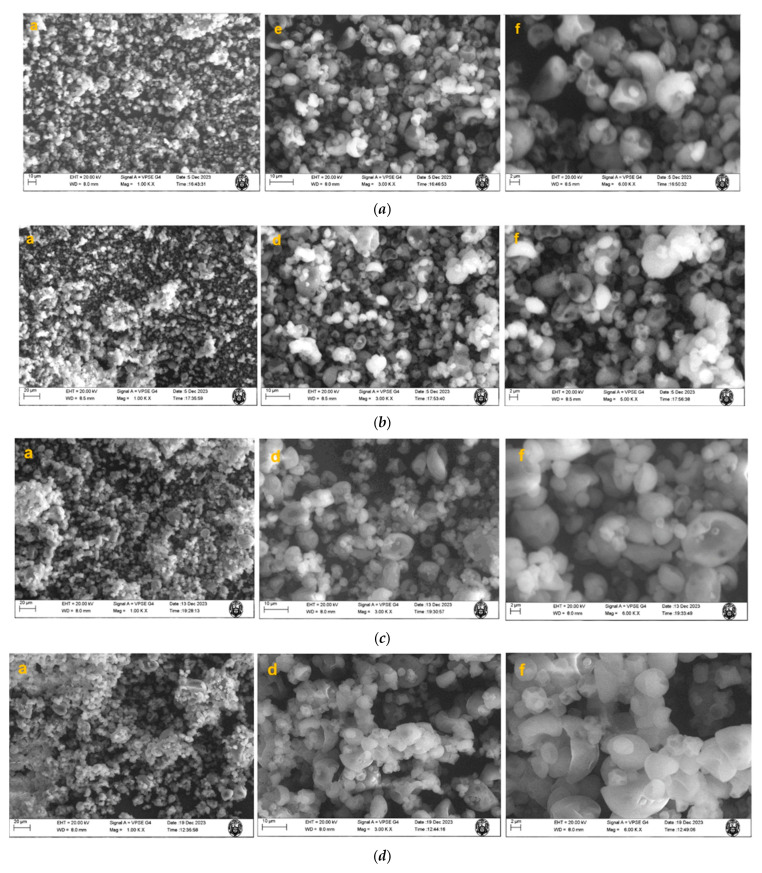
SEM micrographs of the co-microencapsulation of formulations: (***a***) 2 (CP + SIOPH + GA + MD); (***b***) 4 (CP + SIOPH + GOE (200 ppm) + GA + MD); (***c***) 6 (CP + SIOPH + ACHPE (200 ppm) + GA + MD); (***d***) 8 (CP + SIOPH + BHT (200 ppm) + GA + MD). EHT: electronic high voltage. WD: working distance. Mag: magnification.

The morphological analysis performed by scanning electron microscopy (SEM) (Figure 2) showed that the co-microcapsules presented quasi-spherical shapes collapsed with cracks and high agglomeration, which may be due to the high content of surface oil or moisture causing agglomeration of the powder particles [51,52]. Spherical-shaped microcapsules with antioxidants from camu camu skins and BHT were obtained in previous studies, which offered greater oxidative protection to sacha inchi oil (*Plukenetia huayllabambana*) [23]. Other authors observed the presence of spherical particles without cracks or pores in the microencapsulation of chia and fish oil [53,54,55].

### 3.10. Oxidative Stability

Table 11 shows the shelf life values for the eight formulations. Formulation 3 (CP + SIOPH + GOE (200 ppm) + GA) with grape orujo extract had the longest shelf life (4908 ± 184 h), followed by formulation 5 (PC + SIOPH + ACHPE (200 ppm) + GA) (4740 ± 59 h) with aji charapita pepper extract and formulation 1 (PC + SIOPH + GA) (4251 ± 95 h) without antioxidants. These values were higher than those reported by Chasquibol et al. [23], with a shelf life of 3116 h for SIOPH microcapsules with camu camu skin extracts encapsulated with GA + MD + WPI. It was also observed that the formulations containing only GA (formulations 1, 3, 5 and 7) presented higher oxidative stability (4908 ± 184–3579 ± 47 h) than the formulations with GA + MD (1896 ± 51–1334 ± 40 h). Thus, GA, in combination with cushuro polysaccharide, better protected against the lipid oxidation of sacha inchi oil (*Plukenetia huayllabambana*). 

The longest induction times were obtained for formulations 3 (CP + SIOPH + GOE (200 ppm) + GA) (49.68 ± 0.73 h), 5 (CP + SIOPH + ACHPE (200 ppm) + GA) (47.64 ± 0.16) and 1 (CP + SIOPH + GA) (42.02 ± 1.11 h). Induction periods (IP) and stability times tended to halve for each 10 °C increase in temperature due to the high unsaturation of sacha inchi oil [55].

## 4. Conclusions

The co-microencapsulation of cushuro (*Nostoc sphaericum*) polysaccharide with natural antioxidant extracts from grape orujo and aji charapita pepper showed better oxidative stability and a longer shelf life than microcapsules with the commercial antioxidant BHT at 200 ppm. The fatty acid composition (ω-3), the major component in sacha inchi oils, remained stable in all formulations. The same was true for sterols, and in the case of tocopherols, a 19% loss was observed in formulation 1 (PC + SIOPH + GA). The addition of antioxidants did not influence the maintenance of ω-3 acids, total sterols or tocopherols. Formulations with grape orujo extract showed higher oxidative stability (4908 ± 184 h), antioxidant capacity (4835.33 ± 40.02 µg Trolox/g ms) and higher total phenolic content (960.11 ± 53. 59 µg GAE/g ms) than the other formulations, so grape orujo extracts have a greater efficacy than aji charapita pepper and can be used as a replacement for the commercial antioxidant BHT to reduce oxidation in sacha inchi oil (*Plukenetia huayllabambana*). The formulations studied can be industrially useful due to their functional properties and technical feasibility.

## Figures and Tables

**Table 1:** Formulations with cushuro (*Nostoc sphaericum*) polysaccharide and sacha inchi (*Plukenetia huayllabambana*) oil.

Samples	Formulation of Cushuro (*Nostoc sphaericum*) Polysaccharide
1	CP (0.35%) + SIOPH + GA (21.65%)
2	CP (0.40%) + SIOPH + GA (21.60%)
3	CP (0.45%) + SIOPH + GA (21.55%)
4	CP (0.35%) + SIOPH + GA (10.5%) + MD (21.65%)
5	CP (0.40%) + SIOPH + GA (10%) + MD (21.60%)
6	CP (0.45%) + SIOPH + GA (9.0%) + MD (21.55%)

CP: cushuro polysaccharide (*Nostoc sphaericum*); SIOPH: sacha inchi oil (*Plukenetia huayllabambana*); GA: gum arabic; MD: maltodextrin.

**Table 2:** Co-microencapsulation formulations of cushuro (*Nostoc sphaericum*) polysaccharide.

Sample	Formulation of Cushuro (*Nostoc sphaericum*) Polysaccharide with Antioxidant Extracts
1	CP + SIOPH + GA
2	CP + SIOPH + GA + MD
3	CP + SIOPH + GOE (200 ppm) + GA
4	CP + SIOPH + GOE (200 ppm) + GA + MD
5	CP + SIOPH + ACHPE (200 ppm) + GA
6	CP + SIOPH + ACHPE (200 ppm) + GA + MD
7	CP + SIOPH + BHT (200 ppm) + GA
8	CP + SIOPH + BHT (200 ppm) + GA + MD

CP: cushuro polysaccharide (*Nostoc sphaericum*); SIOPH: sacha inchi oil (*Plukenetia huayllabambana*); GOE: grape orujo (*Vitis vinifera* L.) extract; ACHPE: aji charapita pepper (*Capsicum frutences*) extract; BHT: butyl hydroxytoluene; GA: gum arabic; MD: maltodextrin.

**Table 3:** Viscosity of cushuro (*Nostoc sphaericum*) polysaccharide formulations with sacha inchi (*Plukenetia huayllabambana*) oil at 25 °C.

Sample	Formulation	Viscosity (cp)
1	CP (0.35%) + SIOPH + GA (21.65%)	103.82 ± 0.84
2	CP (0.40%) + SIOPH + GA (21.60%)	310.14 ± 0.87
3	CP (0.45%) + SIOPH + GA (21.55%)	356.74 ± 2.58
4	CP (0.35%) + SIOPH + GA (10.5%) + MD (21.65%)	110.47 ± 3.21
5	CP (0.40%) + SIOPH + GA (10%) + MD (21.60%)	308.47 ± 3.64
6	CP (0.45%) + SIOPH + GA (9.0%) + MD (21.55%)	374.5 ± 17.45

Results are expressed as averages ± SD (*n* = 3).

**Table 4:** Moisture content (%) and peroxide index (PI) of co-microencapsulated formulations with cushury polysaccharide (*Nostoc sphaericum*), antioxidant extracts and sacha inchi oil (*Plukenetia huayllabambana*).

Formulation	Co-Microencapsulation of the Cushuro (*Nostoc sphaericum*) Polysaccharide	Moisture Content (%)	Peroxide Index (meq O_2_/Kg oil)
1	CP+SIOPH + GA	4.15 ± 0.62	3.98 ± 0.01
2	CP + SIOPH + GA + MD	4.04 ± 0.09	3.85 ± 0.13
3	CP + SIOPH + GOE (200 ppm) + GA	4.17 ± 0.37	3.04 ± 0.10
4	CP + SIOPH + GOE (200 ppm) + GA + MD	4.71 ± 0.01	3.44 ± 0.32
5	CP + SIOPH + ACHPE (200 ppm) + GA	4.53 ± 0.13	3.48 ± 0.30
6	CP + SIOPH + ACHPE (200 ppm) + GA + MD	4.64 ± 0.03	3.62 ± 0.30
7	CP + SIOPH + BHT (200 ppm) + GA	4.31 ± 0.33	3.50 ± 0.28
8	CP + SIOPH + BHT (200 ppm) + GA + MD	4.28 ± 0.13	3.66 ± 0.11

Results are expressed as averages ± SD (*n* = 3).

**Table 5:** Total phenolic content (TPC), surface phenolic content (SPC), efficiency (% EEP), antioxidant activity (DPPH) and inhibition (%) of co-microencapsulation of cushuro (*Nostoc sphaericum*) polysaccharide with antioxidant extracts and sacha inchi (*Plukenetia huayllabambana*) oil.

Co-Microencapsulation of the Cushuro (*Nostoc sphaericum*) Polysaccharide	TPC (µg GAE/g ms)	SPC (µg GAE/g ms)	EEP (%)	DPPH (µg Trolox/g ms)	Inhibition (%)
**Formulations**					
1	300 ± 29	12 ± 2.4	96 ± 0.6	3169 ± 35	25 ± 0.9
2	257 ± 12	33 ± 5.3	87 ± 1.6	2523 ± 61	22 ± 0.6
3	960 ± 54	18 ± 2.9	98 ± 0.2	4835 ± 40	37 ± 0.4
4	673 ± 44	18 ± 3.2	97 ± 0.6	3983 ± 40	32 ± 1.1
5	662 ± 77	25 ± 8.2	96 ± 1.5	4313 ± 23	36 ± 1.8
6	490 ± 64	23 ± 3.8	95 ± 1.3	3684 ± 34	30 ± 0.7
7	791 ± 39	20 ± 4.9	98 ± 0.5	4641 ± 11	36 ± 1.0
8	628 ± 50	26 ± 11	96 ± 1.8	3428 ± 11	28 ± 0.9

Results are expressed as averages ± SD (*n* = 3).

**Table 6:** Free oil (%) from the co-microencapsulation of cushuro (*Nostoc sphaericum*) polysaccharide with antioxidant extracts and sacha inchi (*Plukenetia huayllabambana*) oil.

Formulation	Co-Microencapsulation of the Cushuro (*Nostoc sphaericum*) Polysaccharide	Free Oil (%)
1	CP + SIOPH + GA	6.52 ± 0.21
2	CP + SIOPH + GA + MD	8.41 ± 0.23
3	CP + SIOPH + GOE (200 ppm) + GA	8.27 ± 0.08
4	CP + SIOPH + GOE (200 ppm) + GA + MD	7.76 ± 0.14
5	CP + SIOPH + ACHPE (200 ppm) + GA	10.79 ± 0.36
6	CP + SIOPH + ACHPE (200 ppm) + GA + MD	14.10 ± 0.49
7	CP + SIOPH + BHT (200 ppm) + GA	9.70 ± 0.66
8	CP + SIOPH + BHT (200 ppm) + GA + MD	8.93 ± 0.40

**Table 7:** Fatty acid composition (%) of co-microencapsulation of cushuro (*Nostoc sphaericum*) polysaccharide with antioxidant extracts and sacha inchi (*Plukenetia huayllabambana*) oil.

Co-Microencapsulation of the Cushuro (*Nostoc sphaericum*) Polysaccharide	C_16:0_	C_16:1_	C_17:0_	C_17:1_	C_18:0_	C_18:1_	C_18:2_	C_20:0_	C_18:3_	C_20:1_	*trans*
SIPHO	4.98 ± 0.04 ^bc^	0.10 ± 0.01	0.07 ± 0.01	0.05 ± 0.01	1.90 ± 0.01 ^c^	8.63 ± 0.03 ^c^	25.91 ± 0.04 ^a^	0.25 ± 0,01	57.86 ± 0.01 ^a^	0.25 ± 0.01	<0.01
1	5.04 ± 0.04 ^abc^	0.08 ± 0.01	0.09 ± 0.01	0.04 ± 0.01	1.93 ± 0.01 ^bc^	8.75 ± 0.07 ^c^	25.91 ± 0.04 ^a^	0.27 ± 0.01	57.63 ± 0.02 ^abc^	0.26 ± 0.01	<0.01
2	5.08 ± 0.06 ^ab^	0.10 ± 0.03	0.10 ± 0.01	0.11 ± 0.03	1.94 ± 0.02 ^ab^	9.33 ± 0.21 ^a^	25.80 ± 0.08 ^a^	0.26 ± 0.01	57.00 ± 0.37 ^bc^	0.28 ± 0.01	<0.01
3	5.14 ± 0.08 ^a^	0.09 ± 0.01	0.09 ± 0.01	nd	1.97 ± 0.04 ^a^	9.15 ± 0.24 ^ab^	25.83 ± 0.02 ^a^	0.26 ± 0.01	57.22 ± 0.31 ^bc^	0.25 ± 0.04	<0.01
4	5.00 ± 0.04 ^bc^	0.07 ± 0.01	0.08 ± 0.01	0.07 ± 0.01	1.91 ± 0.01 ^bc^	8.87 ± 0.15 ^bc^	25.82 ± 0.04 ^a^	0.25 ± 0.01	57.66 ± 0.18 ^abc^	0.27 ± 0.01	<0.01
5	5.01 ± 0.05 ^bc^	0.10 ± 0.01	0.10 ± 0.01	0.11 ± 0.01	1.92 ± 0.01 ^bc^	8.65 ± 0.10 ^bc^	25.87 ± 0.15 ^a^	0.29 ± 0.02	57.66 ± 0.15 ^abc^	0.29 ± 0.01	<0.01
6	4.97 ± 0.02 ^bc^	0.09 ± 0.01	0.08 ± 0.01	0.11 ± 0.06	1.91 ± 0.01 ^bc^	8.80 ± 0.01 ^bc^	25.84 ± 0.0 ^a^	0.25 ± 0.01	57.69 ± 0.01 ^ab^	0.26 ± 0.01	<0.01
7	4.96 ± 0.01 ^c^	0.08 ± 0.01	0.08 ± 0.01	0.12 ± 0.06	1.95 ± 0.01 ^bc^	8.78 ± 0.04 ^c^	25.83 ± 0.01 ^a^	0.25 ± 0.01	57.76 ± 0.01 ^ab^	0.19 ± 0.07	<0.01
8	4.99 ± 0.03 ^bc^	0.11 ± 0.01	0.07 ± 0.00	0.10 ± 0.01	1.90 ± 0.01 ^c^	8.63 ± 0.04 ^c^	25.85 ± 0.01 ^a^	0.25 ± 0.01	57.84 ± 0.09 ^abc^	0.26 ± 0.01	<0.01

nd = not detected. ^a,b,c^ values in the same column with different letters vary significantly at *p* < 0.05. For formulations 1–8, see Table 2.

**Table 8:** Tocopherol composition of the co-microencapsulation of cushuro (*Nostoc sphaericum*) polysaccharide with antioxidant extracts and sacha inchi (*Plukenetia huayllabambana*) oil.

Co-Microencapsulation of the Cushuro (*Nostoc sphaericum*) Polysaccharide	γ-Tocoferol (%)	δ-Tocoferol (%)	Total (mg/kg)	γ/δ
SIOPH	65.56	34.44	1077.8 ± 7.1 ^a^	1.9
Formulation				
1	58.20	41.80	870.5 ± 6.4 ^e^	1.4
2	65.21	34.79	916.2 ± 5.6 ^cd^	1.9
3	66.67	33.33	969.5 ± 7.1 ^b^	2.0
4	61.69	38.31	912.2 ± 7.1 ^d^	1.6
5	66.42	33.58	953.9 ± 8.5 ^b^	2.0
6	67.28	32.72	932.5 ± 7.8 ^c^	2.1
7	66.86	33.14	950.3 ± 9.9 ^b^	2.0
8	65.04	34.96	928.5 ± 8.5 ^cd^	1.9

^a,b,c,d,e^ values in the same column with different letters vary significantly *p* < 0.05. For formulations 1–8, see Table 2.

**Table 9:** Sterol composition of co-microencapsulation of cushuro (*Nostoc sphaericum*) polysaccharide with antioxidant extracts and sacha inchi (*Plukenetia huayllabambana*) oil.

Co-Microencapsulation of the Cushuro (*Nostoc sphaericum*) Polysaccharide	β-Sitosterol (%)	Stigmasterol/Campesterol Ratio	Total (mg/kg)
SIOPH	60.52	4.90	2257.5 ± 17.7 ^a^
Formulation			
1	61.78	6.08	1854.0 ± 22.6 ^b^
2	62.72	5.88	2249.3 ± 28.3 ^a^
3	61.70	6.19	2248.5 ± 26.2 ^a^
4	61.13	4.32	2242.5 ± 24.7 ^a^
5	61.90	5.94	2240.0 ± 25.4 ^a^
6	57.86	4.40	2154.0 ± 22.6 ^b^
7	57.59	3.45	2156.5 ± 24.7 ^b^
8	58.09	6.27	2124.0 ± 28.2 ^b^

^a,b^ values in the same column with different letters vary significantly at *p* < 0.05. For formulations 1–8, see Table 2.

**Table 10:** Solubility (%) and particle size distribution of co-microencapsulation of cushuro polysaccharide with antioxidant extracts and sacha inchi (*Plukenetia huayllabambana*) oil.

Co-Microencapsulation of the Cushuro (*Nostoc sphaericum*) Polysaccharide	% Solubility	D [4,3] (µm)	Span	Volume Distribution (µm)		
				D10 (v,01)	D50 (v,01)	D50 (v,01)
1	79.73 ± 0.30	10.61 (3.04)	1.473	2.43 (0.30)	4.80 (0.89)	9.62 (1.95)
2	81.18 ± 0.45	9.59 (2.66)	1.137	2.91 (0.35)	5.09 (0.82)	8.70 (1.72)
3	84.62 ± 0.69	7.55 (2.15)	1.193	2.19 (0.22)	3.77 (0.60)	6.69 (1.26)
4	87.22 ± 0.63	7.71 (2.12)	1.169	2.68 (0.32)	4.31 (0.63)	7.72 (1.41)
5	82.41 ± 0.57	11.21 (3.33)	1.449	2.95 (0.26)	5.38 (0.89)	10.75 (2.14)
6	86.02 ± 0.87	9.27 (2.64)	1.329	2.73 (0.31)	4.64 (0.78)	8.90 (1.72)
7	83.51 ± 0.47	10.86 (3.18)	1.55	2.14 (0.18)	4.27 (0.75)	8.76 (1.70)
8	85.47 ± 0.47	11.71 (3.11)	1.32	2.95 (0.37)	4.90 (0.77)	9.42 (1.79)

**Table 11:** Shelf life of formulations of co-microencapsulation of cushuro polysaccharide with antioxidant extracts and sacha inchi oil (*Plukenetia huayllabambana*).

Co-Microencapsulation of the Cushuro (*Nostoc sphaericum*) Polysaccharide	Induction Time (h)				Extrapolated Shelf Life a 25 °C (h)
	70 °C	80 °C	90 °C	100 °C	
1	42.02 ± 1.11	18.26 ± 0.47	7.12 ± 0.04	3.63 ± 0.11	4251 ± 95
2	32.62 ± 0.24	13.62 ± 0.33	6.41 ± 0.04	2.97 ± 0.06	2913 ± 178
3	41.68 ± 0.73	24.58 ± 0.23	8.51 ± 0.34	3.84 ± 0.17	4908 ± 184
4	25.82 ± 0.10	13.40 ± 0.38	5.72 ± 0.07	2.76 ± 0.01	1896 ± 51
5	47.64 ± 0.16	19.74 ± 0.19	7.49 ± 0.13	4.08 ± 0.06	4740 ± 59
6	36.08 ± 0.16	15.93 ± 0.44	6.19 ± 0.35	3.34 ± 0.09	3212 ± 151
7	36.28 ± 0.27	16.52 ± 0.24	7.69 ± 0.28	3.44 ± 0.01	3579 ± 47
8	18.70 ± 0.01	9.45 ± 0.06	3.74 ± 0.08	2.01 ± 0.03	1334 ± 40

The results are expressed as averages ± SD (*n* = 3).

## Data Availability

Data are contained within the manuscript.
